# Relative expression of genes of terpene metabolism in different tissues of *Artemisia annua *L

**DOI:** 10.1186/1471-2229-11-45

**Published:** 2011-03-09

**Authors:** Linda Olofsson, Alexander Engström, Anneli Lundgren, Peter E Brodelius

**Affiliations:** 1 School of Natural Sciences, Linnaeus University, SE-39182 Kalmar, Sweden

## Abstract

**Background:**

Recently, *Artemisia annua *L. (annual or sweet wormwood) has received increasing attention due to the fact that the plant produces the sesquiterpenoid endoperoxide artemisinin, which today is widely used for treatment of malaria. The plant produces relatively small amounts of artemisinin and a worldwide shortage of the drug has led to intense research in order to increase the yield of artemisinin. In order to improve our understanding of terpene metabolism in the plant and to evaluate the competition for precursors, which may influence the yield of artemisinin, we have used qPCR to estimate the expression of 14 genes of terpene metabolism in different tissues.

**Results:**

The four genes of the artemisinin biosynthetic pathway (amorpha-4,11-diene synthase, amorphadiene-12-hydroxylase, artemisinic aldehyde ∆11(13) reductase and aldehyde dehydrogenase 1) showed remarkably higher expression (between ~40- to ~500-fold) in flower buds and young leaves compared to other tissues (old leaves, stems, roots, hairy root cultures). Further, dihydroartemisinic aldehyde reductase showed a very high expression only in hairy root cultures. Germacrene A and caryophyllene synthase were mostly expressed in young leaves and flower buds while *epi*-cedrol synthase was highly expressed in old leaves. 3-Hydroxy-3-methyl-glutaryl coenzyme A reductase exhibited lower expression in old leaves compared to other tissues. Farnesyldiphosphate synthase, squalene synthase, and 1-deoxy-D-xylulose-5-phosphate reductoisomerase showed only modest variation in expression in the different tissues, while expression of 1-deoxy-D-xylulose-5-phosphate synthase was 7-8-fold higher in flower buds and young leaves compared to old leaves.

**Conclusions:**

Four genes of artemisinin biosynthesis were highly expressed in flower buds and young leaves (tissues showing a high density of glandular trichomes). The expression of dihydroartemisinic aldehyde reductase has been suggested to have a negative effect on artemisinin production through reduction of dihydroartemisinic aldehyde to dihydroartemisinic alcohol. However, our results show that this enzyme is expressed only at low levels in tissues producing artemisinin and consequently its effect on artemisinin production may be limited. Finally, squalene synthase but not other sesquiterpene synthases appears to be a significant competitor for farnesyl diphosphate in artemisinin-producing tissues.

## Background

The genus *Artemisia*, belonging to the Asteraceae family, contains a large number of aromatic plants. During the latest decades, *A. annua *L. (annual or sweet wormwood) has received increasing attention due to the fact that the plant produces the sesquiterpenoid endoperoxide artemisinin, which today is widely used for the treatment of malaria [1]. The plant produce relatively small amounts of artemisinin and a worldwide shortage of the drug has led to intense research in order to increase the yield of artemisinin in the plant or to develop alternative methods of artemisinin production [2]. Artemisinin and a number of other terpenoids are produced in glandular secretory trichomes present on aerial surfaces of the plant. The glandular trichomes are 10-cell structures with three pairs of secretory cells [3]. The apical cells are transparent proplastid- or leucoplast-containing cells, and the two cell pairs below the apical cells, the sub-apical cells, are green chloroplast-containing cells. The substances that are produced in these cells are excreted into, and stored in the subcuticular sac, which is covering the secretory cells [3]. This has led to the focus on these structures when studying genes involved in terpene biosynthesis in general and for artemisinin biosynthesis in particular.

The major components of the essential oil from *A. annua *are mono- and sesquiterpenes produced in trichomes [4]. The large number of structurally different terpenoids indicates that several mono- and sesquiterpene synthases are expressed in *A. annua*. In fact, so far five different sesquiterpene synthases (*epi*-cedrol synthase (ECS) [5], amorpha-4,11-diene synthase (ADS) [6]; β-caryophyllene synthase (CPS) [7], germacrene A synthase (GAS) [8] and β-farnesene synthase (BFS) [9] have been cloned from the plant. However, it is likely that additional sesquiterpene synthases are expressed in *A. annua *since other classes of sesquiterpenes such as eudesmanes (*e.g. *β-selinene), guaianes (*e.g. *α-guaiene), longipinanes (*e.g. *β-longipinene) and santalanes (*e.g. *α-santalol) have been isolated from the plant [10]. All these sesquiterpene synthases may compete for the same substrate, farnesyl diphosphate (FDP), which is a precursor of artemisinin. Consequently, the activity of various sesquiterpene synthases may influence the yield of artemisinin in the plant. In addition, FDP is used for the synthesis of squalene, which is the precursor of sterols and triterpenes.

**Figure 1:**
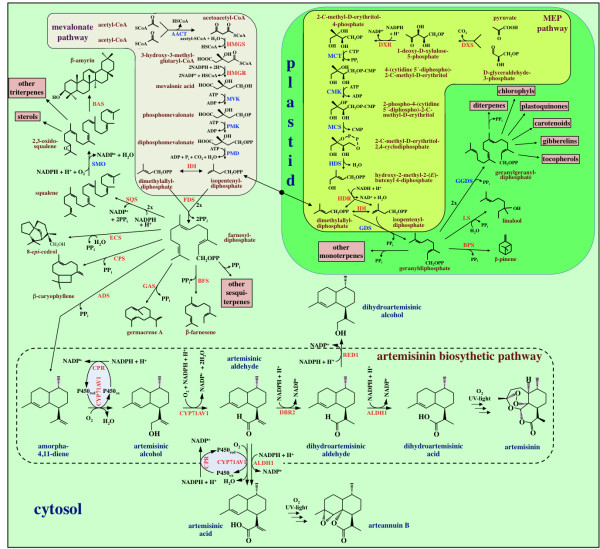
**Summary of terpene metabolism in *Artemisia annua***. Enzymes in red have been cloned from *A. annua*. The Genbank accession numbers are given after each cloned enzyme. ***Cytosol***: AACT: acetoacetyl-CoA thiolase; ADS: amorpha-4,11-diene synthase (AF138959); ALDH1: aldehyde dehydrogenase 1 (FJ809784); BAS: β-amyrin synthase (EU330197); BFS: β-farnesene synthase (AY835398); CPR: cytochrome P450 reductase (EF197890); CPS: β-caryophyllene synthase (AF472361); CYP71AV1: amorphadiene-12-hydroxylase (DQ453967); DBR2: artemisinic aldehyde ∆11(13) reductase (EU704257); ECS: *epi*-cedrol synthase (AJ001539); FDS: farnesyl diphosphate synthase (U36376); GAS: germacrene A synthase (DQ447636); HMGR: 3-hydroxy-3-methyl-glutaryl coenzyme A reductase (AF142473); HMGS: 3-hydroxy-3-methyl-glutaryl coenzyme A synthase (GQ468550); IDI: isopentenyl diphosphate isomerase (DQ666334); MVK: mevalonate kinase; PMD: diphosphomevalonate decarboxylase; PMK: phosphomevalonate kinase; RED1: dihydroartemisinic aldehyde reductase (GU167953); SMO: squalene monooxygenase; SQS: squalene synthase (AY445505). ***Plastid***: BPS: β-pinene synthase (AF276072); CMK: 4-cytidine 5'-diphospho-2-C-methyl-D-erythritol kinase; DXR: 1-deoxy-D-xylulose-5-phosphate reductoisomerase (AF182287); DXS: 1-deoxy-D-xylulose-5-phosphate synthase (AF182286); GGDS: geranylgeranyl diphosphate synthase; GDS: geranyl diphosphate synthase; HDR: hydroxy-2-methyl-2-(*E*)-butenyl 4-diphosphate reductase (EU332141); HDS; hydroxy-2-methyl-2-(*E*)-butenyl 4-diphosphate synthase (FJ479720); IDI: isopentenyl diphosphate isomerase (DQ666334); LS: linalool synthase (AF154125); MCT: 2-*C*-methyl-D-erythritol-4-(cytidyl-5-diphosphate) transferase; MCS: 2-C-methyl-D-erythritol-2,4-cyclodiphosphate synthase.

Figure 1 shows the biosynthetic pathway leading to artemisinin as it is understood today along with other pathways of terpene metabolism in *A. annua*. The first committed step in artemisinin biosynthesis is the conversion of FDP to amorpha-4,11-diene by ADS [6,11]. In the following step, amorpha-4,11-diene is hydroxylated to yield artemisinic alcohol. This reaction is catalyzed by a cytochrome P450 dependent hydroxylase (CYP71AV1) [12]. This enzyme can also oxidize the alcohol to artemisinic aldehyde and then further on to artemisinic acid [12]. It has long been assumed that artemisinic acid is a direct precursor of artemisinin. However, recent feeding experiments with artemisinic acid [13] and dihydroartemisinic acid [14] have shown that the latter substance is the precursor of artemisinin. Dihydroartemisinic acid is formed from artemisinic aldehyde in two steps via dihydroartemisinic aldehyde. The reduction is catalyzed by artemisinic aldehyde Δ11(13) reductase (DBR2) [15] and the oxidation to the acid by aldehyde dehydrogenase 1 (ALDH1) [16]. It has not been fully evaluated if the CYP71AV1 enzyme can catalyze the oxidation of dihydroartemisinic aldehyde to the corresponding acid [16,17].

The conversion of dihydroartemisinic acid to artemisinin is believed to be a non-enzymatic spontaneous reaction [14]. In a similar way, artemisinic acid is converted to arteannuin B as indicated in Figure 1[13].

Recently, a dihydroartemisinic aldehyde reductase (RED1) has been cloned from *A. annua *[18]. This enzyme can potentially convert dihydroartemisinic aldehyde into dihydroartemisinic alcohol, a substance that appears to be a "dead end product", thereby affecting the yield of artemisinin in a negative way.

The genetic variation within *A. annua *appears to be high. At least two chemotypes with different compositions of the essential oil during the vegetative period have been described [19]. One chemotype shows high content of dihydroartemisinic acid and artemisinin, while the second chemotype shows high content of artemisinic acid and arteannuin B but low amounts of artemisinin. According to previous investigations, there is no conversion *in planta *of artemisinic acid to dihydroartemisinic acid or *vice versa *[13]. It has been suggested that arteannuin B can be converted to artemisinin *in planta *[20].

In order to increase our understanding of terpene metabolism in the plant *A. annua *and to evaluate the competition for precursors, which may influence the yield of artemisinin in the plant, we have used qPCR to estimate the expression of genes of terpene metabolism in different plant tissues. cDNA made from flower buds, young leaves, old leaves, stems, roots and hairy roots has been used as templates for the qPCR studies. In total, 14 different transcripts have been monitored using three reference genes. The different tissues have also been studied by fluorescence microscopy to determine trichome densities.

## Results and Discussion

### Trichomes

**Figure 2:**
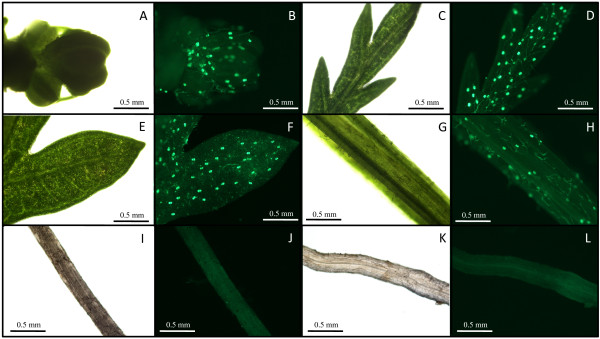
**Light and fluorescence microscopy of various tissues from *Artemisia annua***. A and B: Flower bud; C and D: young leaf; E and F: old leaf; G and H: stem; I and J: root; K and L: hairy root.

The glandular trichomes of *A. annua *have been extensively studied and it is well established that the production of specific terpenoids, including artemisinin, takes place within the secretory cells of such trichomes [21,22]. Furthermore the yield of terpenoids in various plants is highly dependent on trichome abundance [23]. In order to investigate the differences in trichome density on different plant tissues, glandular trichomes of *A. annua *were visualized by fluorescence microscopy (Figure 2) and found in all aerial tissues of the plant such as flower buds (panels A and B), leaves (panels C-F), stems (panels G and H) at different densities, but as expected not on roots (panels I and J) or hairy root cultures of *A. annua *(panels K and L).

From Figure 2, it is evident that young leaves (panels C and D) carry considerably higher density of trichomes than older leaves (panels E and F). All these tissues were taken from 5-6 months old plants with flower buds, which had been induced by a 21 days short day treatment (8 h day). We have estimated, by counting trichomes, that on young leaves there are around 300±40 trichomes/cm^2 ^while the corresponding number for old leaves was 130 ± 25 trichomes/cm^2^. The original number of trichomes seem to be spread out on a larger surface as the leaf expands with age.

### Gene expression

In order to examine the gene expression of 14 enzymes of terpene metabolism and three reference transcripts by qPCR, 18 primer pairs were synthesized as listed in Table 1. The efficiency of these primer pairs was calculated from the qPCR experiments and was found to be between 1.82 and 1.93 as summarized in Table 1.

**Table 1:** Efficiency and nucleotide sequences of primers used in qPCR

Transcript*	Calculated primer efficiency	Forward Primer Sequence	Reverse Primer Sequence	Fragment size (bp)
**β-actin**	**1.91**	**5'-CCAGGCTGTTCAGTCTCTGTAT-3'**	**5'-CGCTCGGTAAGGATCTTCATCA-3'**	**180**

ADS	1.88	5'-GGGAGATCAGTTTCTCATCTATGAA-3'	5'-CTTTTAGTAGTTGCCGCACTTCTT-3'	95

ALDH1	1.82	5'-CATCGGAGTAGTTGGTCACAT-3'	5'-GTTTCTGACCCAAATCCAGGTTGA-3'	120

**CPR**	**1.90**	**5'-GCTCGGAACAGCCATCTTATTCTT-3'**	**5'-GAAGCCTTCTGAGTCATCTTGTGT-3'**	**174**

CPS	1.85	5'-CAACGATGTAGAAGGCTTGCTTGA-3'	5'-GTAGATAGTGTTGGGTTGGTGTGA-3'	150

CYP71AV1	1.83	5'-CGAGACTTTAACTGGTGAGATTGT-3'	5'-CGAAGCGACTGAAATGACTTTACT-3'	144

DBR2	1.91	5'-GCGGTGGTTACACTAGAGAACTT-3'	5'-ATAATCAAAACTAGAGGAGTGACCC-3'	228

DBR2/OPR3	1.84	5'-ATCATCAACAAGCAAGCCCATTTCAAA-3'	5'-CGATAGTCTTCAACCACCTCTAGTA-3'	125

DXR	1.92	5'-GGTGATGAAGGTGTTGTTGAGGTT-3'	5'-AGGGACCGCCAGCAATTAAGGT-3'	160

DXS	1.92	5'-GTGCTTCCAGACCGTTACATTGA-3'	5'-AGCCTCTCGTGTTTGCCCAAGGT-3'	120

ECS	1.86	5'-GCAACAAGCCTACGAATCACTCAA-3'	5'-CGTGAAAAATTAAGGACCCTCATAG-3'	126

FDS1/FDS2	1.93	5'-ATCTGCCCTTGGTTGGTGTATTGA-3'	5'-GTTGCCCTCTGCGTGTATGAGA-3'	92

GAS	1.83	5'-CTCGTTACTCCTTGGCAAGAATCAT-3'	5'-GCTCCATAGCACTAATATCCCACTT-3'	147

HDR	1.86	5'-TCAGGAGCGACAAGATGCTATGTA-3'	5'-AGTGTGAGGTGTTGCTTGAGTTGA-3'	95

HMGR	1.83	5'-GGGCGTGGAAAATCTGTTGTGTTC-3'	5'-GAACCAGCAATAGCAGAACCAGTAA-3'	136

**PAL**	**1.87**	**5'-ATCGGGAAACTCATGTTCGCTCAA-3'**	**5'-AACTTGGGTTACGTCCACCAGAAA-3'**	**97**

RED1	1.92	5'-TGTCAACTGTGTCCATCCAGGTT-3'	5'-ACCATCATCGGGCAACAAAGCAA-3'	118

SQS	1.82	5'-GACCAGTTCCACCATGTTTCTACT-3'	5'-GCTTTGACAACCCTATTCCAACAAG-3'	190

The mean efficiency of the amplicons was calculated by the program Linreg v. 12.1 based on the log linear phase of the amplification curve. The transcripts in bold were used as reference genes in the calculation of expression ratios.

* ADS, amorpha-4,11-diene synthase; ALDH1, aldehyde dehydrogenase 1; CPR, cytochrome P450 reductase; CPS, β-caryophyllene synthase; CYP71AV1, amorphadiene-12-hydroxylase; DBR2, artemisinic aldehyde Δ11(13) reductase; DXR, 1-deoxy-D-xylulose 5-phosphate reductase; DXS, 1-deoxy-D-xylulose 5-phosphate synthase; ECS, *epi*-cedrol synthase; FDS, farnesyl diphosphate synthase; GAS, germacrene A synthase; HDR, hydroxy-2-methyl-2-(*E*)-butenyl 4-diphosphate reductase; HMGR, 3-hydroxy-3-methyl-glutaryl-CoA reductase; OPR3, 12-oxophytodienoate reductase; PAL, phenylalanine ammonia lyase; RED1, dihydroartemisinic aldehyde reductase; SQS, squalene synthase.

#### Relative expression of genes of terpene metabolism in different tissues

**Figure 3:**
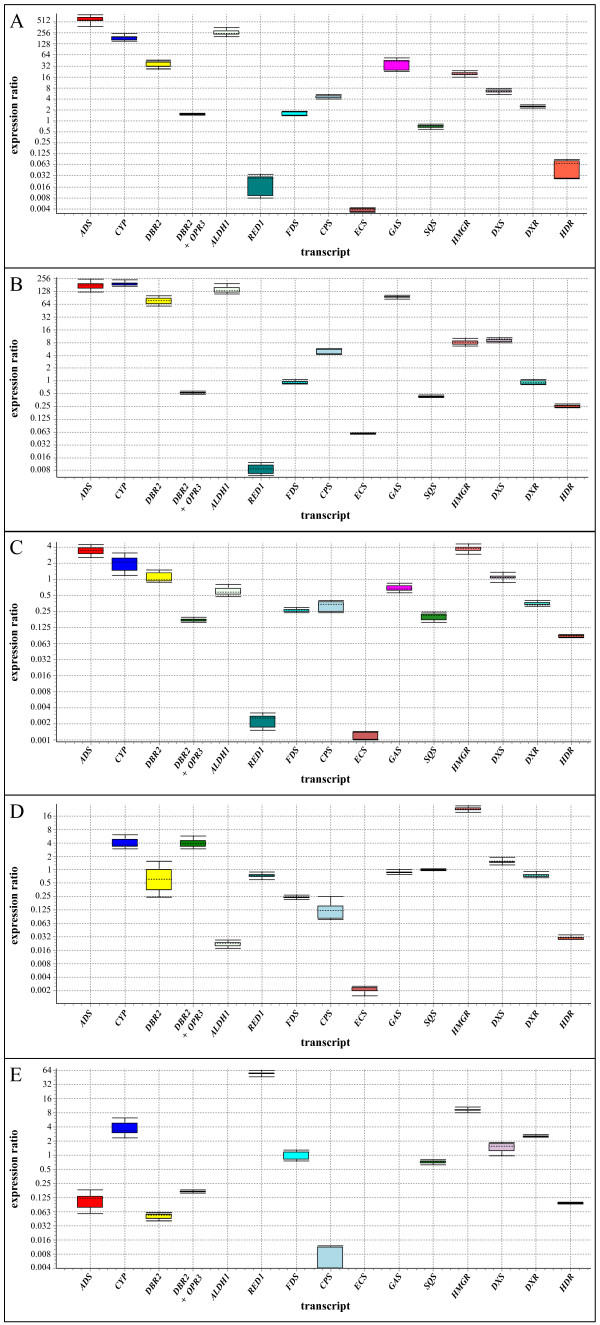
**Expression of genes involved in terpene metabolism in different tissues of *Artemisia annua***. Expression levels were measured through qPCR using the primers listed in Table 1. The expression ratios were calculated relative to the expression in old leaves. A: Flower buds; B: young leaves; C: stems; D: roots; E: hairy roots. All graphs represent the values of two independent qPCR runs with cDNA prepared from different plants. Sample triplicates were used in all qPCR runs. Expression ratios are illustrated by box-and-whisker plots. Boxes above expression ratio 1 represent higher gene expression levels compared to old leaves. Note that the expression ratio scale is logarithmic and not the same in all graphs.

The gene expression of the 14 enzymes of terpene metabolism was studied in different tissues of *A. annua *and the results are summarized in Table 2 and Figure 3. Transcripts of all the genes studied could be detected in all tissues at different amounts using qPCR. The only exception was that no ADS could be detected in roots. In Figure 3, expression levels in the different tissues are all compared separately to old leaves, which were arbitrary chosen as reference tissue. The results of the different experiments are presented below.

**Table 2:** Average measured *C*_T_-values

Transcript*	Flower buds	Young leaves	Old leaves	Stems	Roots	Hairy roots
**β-actin**	**23.8**	**22.3**	**23.4**	**21.6**	**22.9**	**21.1**

**β-actin^#^**	**20.8**	**22.1**	**22.5**	**20.7**	**24.4**	**20.3**

ADS	23.3	22.2	29.9	30.0	n.d.	35.9

ALDH1	25.8	27.7	31.7	34.3	37.4	39,5

**CPR**	**21.5**	**21.0**	**19.9**	**19.5**	**20.7**	**21.0**

CPS	27.9	27.5	31.4	30.3	35.5	37,7

CYP71AV1	26.9	24.2	33.8	30.6	31.3	30.7

DBR2^#^	19.9	20.1	27.2	26.4	27.9	29.6

DBR2+OPR3	23.8	23.0	24.4	23.0	23.8	25.1

DXR	24.6	24.7	24.8	23.8	25.6	23.4

DXS	21.9	20.8	23.0	21.1	22.6	23.1

ECS	28.1	25.7	20.6	29.1	30.5	35.6

FDS1/FDS2	24.6	23.5	23.5	23.8	25.3	23.5

GAS	30.6	25.1	32.6	34.2	32.6	34.0

HDR	24.4	22.4	19.7	22.4	25.4	24.3

HMGR	24.6	24.4	28.6	24.6	23.8	24.7

**PAL**	**21.8**	**20.0**	**21.3**	**19.4**	**20.8**	**21.7**

RED1	31.7	30.7	26.0	34.6	25.7	20.9

SQS	26.7	26.5	25.2	25.1	26.5	25.7

Two independent triplet reactions were used for the calculation of average C_T_-values. n.d. = not detected. The rows in bold represent the reference samples. The transcripts labelled with ^# ^were analyzed in a separate experiment.

* ADS, amorpha-4,11-diene synthase; ALDH1, aldehyde dehydrogenase 1; CPR, cytochrome P450 reductase; CPS, β-caryophyllene synthase; CYP71AV1, amorphadiene-12-hydroxylase; DBR2, artemisinic aldehyde Δ11(13) reductase; DXR, 1-deoxy-D-xylulose 5-phosphate reductase; DXS, 1-deoxy-D-xylulose 5-phosphate synthase; ECS, *epi*-cedrol synthase; FDS, farnesyl diphosphate synthase; GAS, germacrene A synthase; HDR, hydroxy-2-methyl-2-(*E*)-butenyl 4-diphosphate reductase; HMGR, 3-hydroxy-3-methyl-glutaryl-CoA reductase; OPR3, 12-oxophytodienoate reductase; PAL, phenylalanine ammonia lyase; RED1, dihydroartemisinic aldehyde reductase; SQS, squalene synthase.

#### Relative expression of genes encoding HMGR and FDS in different tissues

The mevalonate pathway produces IDP and DMADP for production of different types of terpenoids as summarized in Figure 1. One enzyme of the mevalonate pathway (3-hydroxy-3-methyl-glutaryl-CoA reductase; HMGR) has been studied by qPCR in this paper. Our results show that HMGR was expressed at higher levels in biosynthetically active tissues such as flower buds, young leaves, roots and hairy roots. The *C*_T_-values for this transcript in these tissues were relatively low (23.8-24.7) indicating that HMGR was expressed at relatively high levels (Table 2). HMGR is a rate-limiting enzyme of the mevalonate pathway [24,25], and recently it has been shown that HMGR expression limits artemisinin formation in *A. annua *[26]. The regulation of the mevalonate pathway by HMGR was reflected in the result that around 20- and 8-fold more expression was seen in highly biosynthetically active flower buds and young leaves, respectively, than in old leaves (Figure 3). Stermer *et al. *showed that the HMGR activity is much reduced in mature tissues, correlating well with the results presented here [27]. It is interesting to note that HMGR also was enhanced 20-fold in roots. This might be due to the relatively rapid growth of roots and the requirement for sterols and protective terpenoids [25].

Farnesyl diphosphate synthase (FDS) plays a central role in the conversion of IDP and DMADP, produced by the mevalonate pathway, to various terpenoids such as sterols and sesquiterpenes. A small gene family encodes isoenzymes of FDS in plants. *FDS1 *is a housekeeping gene as it is expressed in all tissues at all developmental stages [28]. It is involved in the synthesis of isoprenoids serving basic plant cell functions such as production of sterols for various membranes. Overexpression of *FDS1 *in *A. annua *results in an increase in artemisinin production [29,30] indicating that it is a rate-limiting enzyme in this pathway. *FDS2 *is often inducible and found in special tissues at particular stages of development [28] and is also involved in phytoalexin biosynthesis. *FDS1 *[31,32] and *FDS2 *(GenBank accession number AF136602) have been cloned from *A. annua*. The two *FDS *genes from *A. annua *show 96.6% nucleotide sequence identity and the segment that was amplified with the primers used (Table 1) is identical in the two genes. Consequently, the expression of both genes was monitored in our qPCR experiments. From Figure 3 it is evident that the total expression of *FDS1/FDS2 *is essentially the same in all tissues, and the relatively small variation observed may be explained by the fact that the housekeeping gene *FDS1 *constitutes a major part of total *FDS1/FDS2 *and that the eventual different level of *FDS2 *expression in different tissues thereby becomes masked.

#### Relative expression of genes of the MEP pathway in different tissues

The MEP pathway produces the precursors for many different terpenoids as shown in Figure 1. Three of the eight enzymes of the MEP pathway, 1-deoxy-D-xylulose 5-phosphate synthase (DXS), 1-deoxy-D-xylulose 5-phosphate reductase (DXR) and hydroxy-2-methyl-2-(*E*)-butenyl 4-diphosphate reductase (HDR), have been studied by qPCR in this paper. Our results show that the expression of DXS was 7-8-fold higher in flower buds and young leaves compared to old leaves (Figure 3). It is well established that DXS, the first enzyme of the MEP pathway, plays a major role in the overall regulation of the pathway [33]. Higher expression of DXS leads to an enhanced production of IDP and DMADP, which may influence the synthesis of terpenoids in the plastid. However, an enhanced DXS activity may also influence the production of artemisinin since it has recently been shown that one C_5_-unit of FDP, the precursor of artemisinin, is produced by the MEP pathway [34]. In the other tissues, the expression of DXS was equal to that in old leaves. DXR and HDR may also have rate-limiting roles for the production of IDP and DMADP [33]. The expression of DXR appears to be somewhat enhanced in flower buds and hairy roots but lower in stems and roots. The expression of HDR was 10-30-fold higher in old leaves than in the other tissues.

#### Relative expression of genes of artemisinin biosynthesis in different tissues

During the last decade, a number of enzymes of artemisinin biosynthesis have been cloned and a putative pathway has been constructed as outlined in Figure 1. We have monitored the expression of four genes (ADS, CYP71AV1, DBR2 and ALDH1) involved in the conversion of FDP to dihydroartemisinic acid, which is a late precursor of artemisinin [14]. We have also included RED1 in these studies as it has been suggested that this enzyme may have an influence on the yield of artemisinin by withdrawing dihydroartemisinic aldehyde from further conversion to artemisinin [18].

The first committed enzyme of artemisinin biosynthesis is ADS [11], which has been cloned by several investigators [6,35-37]. The next enzyme, CYP71AV1, was cloned by two groups independently [12,38]. The following two enzymes, DBR2 and ALDH1, were cloned and characterized by Zhang *et al. *[15] and Teoh *et al. *[16], respectively. Finally, RED1 was cloned and characterized by Rydén *et al. *[18].

In Table 3 we summarize previous studies of gene expression of enzymes involved in artemisinin biosynthesis in *A. annua*. The specific expression of CYP71AV1, DBR2 and ALDH1 in glandular trichomes of *A. annua *has been shown by PCR, and ADS has been shown to be expressed in glandular trichomes by immuno gold staining in combination with silver enhancement [39]. The relative expression of these different genes cannot be compared since the data in Table 3 is from several different studies and on different varieties of *A. annua*. It may, however, be concluded from Table 3 that the biosynthetic enzymes are expressed in flower buds, leaves and stems that carry glandular trichomes but not in roots.

**Table 3:** Relative gene expression of enzymes of artemisinin biosynthesis in different tissues of *A. annu a*

Transcript	Trichome	Flower	Leaf	Stem	Root	Method	Reference
ADS	n.d.	+++	+++	+++	-	RT-PCR	[37]

CYP71AV1	+++++	+++	+	n.d.	+	RT-PCR	[12]

DBR2	+++++	++	+	n.d.	-	qRT-PCR	[15]

ALDH1	+++++	+++	+	n.d.	-	RT-PCR	[16]

RED1	n.d.	+	++	-	n.d.	RT-PCR	[18]

The results are not directly comparable between enzymes. An estimation based on published figures of the relative level of enzymes in different tissues is indicated with +-signs. - = no expression; n.d. = not determined.

In *A. annua*, DBR2 belongs to a small family of enolate reductases and a second enzyme of this family is 12-oxophytodienoate reductase (OPR3), which in *Arabidopsis *is involved in jasmonic acid biosynthesis [40]. The OPR3-like gene of *A. annua *(Genbank accession number EU848577) shows a very high similarity to DBR2, and in our initial attempt to determine the relative expression of DBR2, a primer pair (see Table 1) with identical nucleotide sequence in the DBR2 and OPR3-like sequences was used. Consequently, the two transcripts were amplified simultaneously. The combined expression of these two genes is essentially the same in flower buds, young leaves and old leaves (Figure 3; panels A and B). However, a 21 bp deletion in the DBR2 sequence was thereafter used to design, by spanning this deletion, a reversed primer that is specific for DBR2. Using this primer, the expression of DBR2 could be determined as discussed below.

The qPCR data presented here clearly demonstrate that the expression levels of ADS, CYP71AV1, DBR2 and ALDH1 were very high in flower buds and young leaves compared to old leaves (Figure 3; panel A and B), *i.e. *~500 and ~150 times more ADS, ~150 and ~150 times more CYP71AV1, ~40 and ~70 times more DBR2 and ~250 and ~130 times more ALDH1 in flower buds and young leaves, respectively. These high expression levels indicate a high capacity to produce artemisinin precursors in these tissues carrying biosynthetically active trichomes. These findings are supported by a study on the induction of enzymes involved in artemisinin biosynthesis by jasmonic acid [41]. It was shown by qPCR that the levels of ADS, CYP71AV1, DBR2 and ALDH1 were around 50, 13, 160 and 55 times higher in isolated trichomes (from flower buds) than in leaves, respectively [41], which once again shows that the genes encoding enzymes of artemisinin biosynthesis are specifically expressed in trichomes.

Even though a 70-150-fold difference in expression of the four genes was observed between young and old leaves, the trichome density on these tissues only differs around three times. Obviously, the relative amount of the biosynthetic enzymes is much higher in young trichomes, and the regulation of expression of these enzymes appears to be linked to the developmental stage of the trichomes.

The qPCR data may be used to estimate the relative amount of transcripts using the 2^-ΔΔ*C*T ^method [42] and the β-actin as reference gene. As an example, we have made such an estimation for flower buds. For this crude estimation, we assume that the number of active sites is proportional to the level of transcription and that the enzymes are working at substrate saturation with an optimal NADPH/NADP^+ ^ratio. Under such conditions the k_cat_-value is a good indicator of the conversion of substrate to product. Consequently, we may calculate the relative turnover potential for the different enzymes. The values were normalized to RED1 (=1) due to its low abundance in flower buds. Using published k_cat_-values for the different enzymes, we may calculate their relative turnover potential *in planta *as summarized in Table 4. For CYP71AV1 no k_cat_-value is available and therefore this enzyme is not included in this estimation. It may be concluded that ADS is a rate-limiting enzyme due to its low k_cat_-value and that the other trichome-specific enzymes DBR2 and ALDH1 (Figure 3) are present in excess assuming that they are acting on the same pool of intermediates. Due to differences in the k_cat_-value, ALDH1 exhibits a 5-fold higher potential conversion of dihydroartemisinic aldehyde as compared to artemisinic aldehyde (Table 4).

**Table 4:** Estimation of relative turnover potential for enzymes of artemisinin biosynthesis using the 2^-ΔΔ*C*T ^method [42]

Enzyme	Substrate	K_m _(μM)	k_cat _(s^-1^)	ΔΔ*C*_T_	Normalized transcript amount relative to *RED1 *2^-ΔΔ*C*T^	relative turnover	Reference for kinetic constants
FDS1	IDP	29.4	0.7	-7.1	137	96	[32]
	GDP	17.2					

ADS	FDP	2	0.004	-8.4	338	1.4	[54]

DBR2	AA	19	2.6	-7.9	239	621	[15]

ALDH1	DHAA	8.8	7.7	-5.9	60	462	[16]
	AA	2.6	1.5	-5.9	60	90	

RED1	DHAA	67	0.28	0.0	1	0.3	[18]

β-Actin was used as reference gene. The C_T_-values for flower buds (Table 2) were used for the calculations. The *RED1 *gene was used for the normalization of values. Substrates: AA, artemisinic aldehyde; AAOH, artemisinic alcohol; AD, amorpha-4,11-diene; DHAA, dihydroartemisinic aldehyde; FDP, farnesyldiphosphate; GDP, geranyldiphosphate; IDP, isopentenyldiphosphate.

From Figure 3, we may conclude that only part of the FDS1/FDS2 activity is present in trichomes and involved in artemisinin biosynthesis. In fact, it may be speculated that FDS1 is a critical enzyme for the yield of artemisinin. If we assume that only one percent of the FDS1 activity is found in trichome cells, the relative turnover potential is in the same range as ADS. This assumption is supported by the fact that upregulation of FDS by genetic transformation of *A. annua *using constitutive promoter resulted in an increased artemisinin production [29] indicating that FDS1 also may be a rate-limiting enzyme in artemisinin biosynthesis. However, due to the relative high potential conversion capacity of DBR2 and ALDH1 it may be assumed that as amorpha-4,11-diene is formed by ADS, it is efficiently converted all the way to dihydroartemisinic acid, which accumulates in this chemotype of *A. annua*. This is supported by the fact that essentially no amorpha-4,11-diene is detected by GC-MS of an hexane extract from this variety of *A. annua *(unpublished). Finally, since dihydroartemisinic acid accumulates in this chemotype, we may conclude that the activity of DBR2 is significantly higher than that of CYP71AV1. The fact that DBR2 is a soluble cytosolic protein while CYP71AV1 is embedded in the ER may influence the metabolic flow and the rate of intracellular transport of intermediates has to be considered. At present, we do not have any information on the transport of intermediates of artemisinin biosynthesis within cells. It would be interesting to study the ratio of DBR2 and CYP71AV1 in the two different chemotypes of *A. annua *since these enzymes are the key enzymes for the formation of dihydroartemisinic acid and artemisinic acid, respectively.

Next it may be concluded that RED1 does not appear to have any significant influence on the biosynthesis of artemisinin, which was suggested by Rydén *et al. *[18] due to a high K_m _for dihydroartemisinic aldehyde, low relative turnover potential and only partial localization to trichomes. Expression of RED1 in flower buds, young leaves and stems was relatively low and just a fraction of that observed in old leaves (Figure 3A-3C). Furthermore, it is interesting to note that a high expression of RED1 was observed in hairy roots of *A. annua*, *i.e. *around 50 times more than in old leaves (Figure 3E). The function of RED1 in hairy roots is not known but in this case the high RED1 activity together with an extremely low ALDH1 expression may influence the yield of artemisinin in hairy roots, which is relatively low [43,44].

Finally, no ADS could be detected in roots of *A. annua *while small amounts were found in hairy roots. Hairy roots produce as mentioned above small amounts of artemisinin. However, ALDH1 was almost not detectable in hairy roots, which may lead to the formation of artemisinic acid and arteannuin B instead of artemisinin. These compounds have been isolated from hairy root cultures of *A. annua *[45].

#### Relative expression of genes of sesquiterpene biosynthesis in different tissues

In addition to ADS, four other sesquiterpene synthases have been cloned from *A. annua*. These enzymes with very similar kinetic properties compete for the FDP available and may therefore influence the production of artemisinin precursors. Three of these sesquiterpene synthases, *i.e. *CPS, ECS and GAS have been monitored by qPCR in this study.

The expression of ECS was much lower in flower buds and young leaves than in old leaves (Figure 3A and 3B), while expression of CPS and GAS was considerably higher in these tissues as compared to old leaves. In old leaves, ECS appears to be the only sesquiterpene synthase highly expressed. The function of ECS in *A. annua *has not yet been established.

The relative amounts of the sesquiterpene synthases has been calculated using the 2^-ΔΔ*C*T ^method [42] for some tissues as summarized in Table 5 using β-actin as reference. The values were normalized to GAS expression in stems (=1) due to its low abundance there. As can be seen in Table 5, ADS is the dominating sesquiterpene synthase in flower buds and young leaves, *i.e*. the tissues where the biosynthesis of artemisinin precursors take place. It may be concluded that ADS is capable to channel a major part of the available substrate into artemisinin biosynthesis. Ma *et al *[2009] showed that upregulation of ADS by genetic transformation of *A. annua *resulted in an increased formation of artemisinin showing that ADS is a rate-limiting enzyme [46].

**Table 5:** Estimation of relative amounts of sesquiterpene synthases and squalene synthase in various tissues of *Artemisia annua *using the 2^-ΔΔ*C*T ^method [42]

	Normalized transcript amount relative to *GAS *2^-ΔΔ*C*T^			
Enzyme	Flower buds	Young leaves	Old leaves	Stems

ADS	8780	6654	69	18

CPS	360	170	24	15

GAS	56	890	11	1

ECS	315	590	43000	34

SQS	830	340	1780	550

The C_T_-values in Table 2 were used for the calculations, and β-actin was used as reference gene. The *GAS *gene in stems was used for the normalization of values.

#### Relative expression of genes of sterol and triterpene biosynthesis in different tissues

A key enzyme in the biosynthesis of sterols and triterpenes is squalene synthase (SQS), which condensates two molecules of FDP to squalene. The expression of SQS varied only modest between different tissues in our qPCR study. SQS is a microsomal enzyme and difficult to purify and characterize. Consequently, there is no reported k_cat_-value for any plant enzyme, but a k_cat _= 3.3 sec^-1 ^has been reported for a soluble recombinant yeast enzyme [47]. The *A. annua *enzyme has been cloned and is similar to the enzymes from *A. thaliana *and *Nicotiana tabacum *[48]. As seen in Table 5, a significant amount of SQS was expressed in the tissues with high artemisinin biosynthesis. Assuming a k_cat_-value for the *A. annua *SQS in the order of 1 sec^-1 ^(= 2 FDPs used per second), results in about 45- and 25-fold higher turnover potential, of FDP to products, for SQS, as compared to the turnover potential of ADS in flower buds and young leaves, respectively. The fraction of SQS expressed in trichome cells may channel part of available FDP into sterols and thereby lower the yield of artemisinin. Zhang *et al. *showed that down-regulation of SQS by hairpin-RNA-mediated gene silencing in *A. annua *resulted in a 3-fold increased artemisinin production [49].

## Conclusions

The aim of our studies was to increase our understanding of terpene metabolism in the plant *A. annua*. The qPCR data presented in this paper demonstrate that four genes of the artemisinin biosynthetic pathway (ADS, CYP71AV1, DBR2 and ALDH1) showed remarkably higher expression (between 40- to 500-fold) in flower buds and young leaves compared to other tissues (old leaves, stems, roots and hairy root cultures) (Figure 3). These high expression levels indicate a much higher capacity to produce artemisinin precursors in flower buds and young leaves, which is partly due to the considerably higher density of trichomes on these tissues (Figure 2).

Our aim was also to evaluate the competition for precursors, which may influence the yield of artemisinin in the plant. The expression of other sesquiterpene synthases was much lower than ADS in tissues producing artemisinin precursors (*i.e. *flower buds and young leaves). Consequently, their influence on artemisinin yield appear to be relatively limited and downregulation of other sesquiterpene synthase(s) will most likely not affect artemisinin production in *A. annua*.

However, squalene synthesis may influence the yield of artemisinin, since the potential utilization of FDP by SQS is most likely higher than that of ADS. The fact that ADS is a cytosolic enzyme and SQS is localized to the ER may be favorable for the sesquiterpene synthase. Further, the ratio of CYP71AV1 and DBR2 may be critical for the type of end product formed; high DBR2 activity will result in an efficient formation of dihydroartemisinic aldehyde, dihydroartemisinic acid and artemisinin while high CYP71AV1 activity may result in artemisinic acid and arteannuin B. A direct comparison of the capacity of these two enzymes is not possible due to the fact that no kinetic data is available for CYP71AV1. However in this chemotype of *A. annua*, the turnover capacity of DBR2 is apparently sufficient to convert most of the artemisinic aldehyde to dihydroartemisinic aldehyde for further conversion to artemisinin. It is notable that the RED1 activity was very low in artemisinin-producing tissues and consequently this enzyme appears not to influence the yield of the artemisinin precursor dihydroartemisinic acid to any significant extent.

## Methods

### Tissue Preparation

	Seeds of *Artemisia annua *L. cv Artemis were obtained from Anamed, Germany (http://www.anamed.net). This variety is a high dihydroartemisinic acid chemotype (the ratio artemisinic acid to dihydroartemisinic acid is 0.36 [50]). Plants were grown under 16 h days and 8 h nights at 22°C to a height of approximately 1 m followed by flower bud induction at 8 h days and 16 h nights at 22°C.

*Artemisia annua *L. hairy root cultures, kindly provided by Dr. Kanyaratt Supaibulwatana, Mahidol University, Bangkok, were grown in MSMO medium (Sigma-Adrich, Stockholm, Sweden) pH 5.8, supplemented with 3% (w/v) sucrose at room temperature on a rotary shaker at 75 rpm.

Flower buds, young leaves, old leaves, stems, roots and hairy roots were collected separately from 5-6 months old plants after induction of flower buds, frozen in liquid nitrogen, ground to a fine powder in a mortar and used for RNA extraction.

### RNA extraction

RNA extraction was performed using Purelink™ Plant RNA Reagent kit (Invitrogen, Carlsbad, California, USA) for small scale RNA isolation according to the manufacturer's instructions. Frozen plant tissue powder (100 mg) was used for each RNA extraction. The RNA was DNase treated using DNase I (Fermentas, St Leo-Roth, Germany) to remove remaining genomic DNA.

### First strand cDNA synthesis

RNA (1 μg) was reverse transcribed using RevertAid™ H Minus-MuLV reverse transcriptase (Fermentas) primed with 0.5 μg oligo(dT)_18 _primer. The RNA was removed from the first strand cDNA by RNase treatment using RNase H (Fermentas) according to the manufacturer's instructions.

### Quantitative PCR (qPCR)

The qPCR was performed using specific primers (Table 1) on a 7500 qPCR equipment (Applied Biosystems, Foster City, USA). First strand cDNA was used as template in 20 μl reactions including 10 μl Power SYBR^®^Green PCR Master Mix (Applied Biosystems) and 2 pmol of each primer. The qPCR cycling was performed at 50 °C (2 min), 95 °C (10 min), 40 cycles at 95 °C (15 s), 60 °C (1 min) and finally a dissociation stage at 95 °C (15 s), 60 °C (1 min), 95 °C (15 s). The dissociation stage was performed to determine the PCR product size and to detect possible primer dimers. Triplets of all samples were run, and a negative control of the Master Mix in addition of primers was performed in all qPCR runs.

Any outliers in the triplets were detected by Grubbs test and removed. The mean efficiency of the amplicons was calculated by the program Linreg v. 12.1 based on the log linear phase of the amplification curve [51]. Baseline corrected data was imported set between cycles 3-15, from the 7500 qPCR software (Applied Biosystems) to the Linreg software. Linreg calculated window of linearity for each amplicon and the efficiency from the curve fit showing highest correlation value between 4-6 points within the window of linearity. The cycle threshold (*C*_T_) values and efficiency values given by the Linreg software were used for further analysis. The BestKeeper software [52] was used to search for stable reference genes among all genes tested. Based on BestKeeper, the genes CPR, β-actin and PAL were selected as reference genes. Relative expression levels were calculated using the REST 2009 software V. 2.0.13 (Qiagen, Hilden, Germany) [53].

### Fluorescence Microscopy

Flower buds, young leaves, old leaves, stems and roots were selected from the same *A. annua *plant and microscopy studies were performed to compare the amount of trichomes on different plant tissue. All micrographs were captured using a Nikon e400 C-SHG1 fluorescence microscope equipped with digital camera, using light microscopy and filter settings for FITC (*λ*_ex _480 nm; *λ*_em _= 535 nm) and Texas Red (*λ*_ex _570 nm; *λ*_em _= 625 nm, images not shown). Autofluorescence was separated in the red and green channels and brightness adjusted using NIS-elements imaging software v. 2.20 (Nikon, Badhoevedorp, The Netherlands). All tissue images have been captured using the same magnification (4x objective; 10x on ocular).

## Abbreviations

AA: artemisinic aldehyde; AAOH: artemisinic alcohol; AD: amorpha-4,11-diene; ADS: amorpha-4,11-diene synthase; ALDH1: aldehyde dehydrogenase 1; CPR: cytochrome P450 reductase; CPS: β-caryophyllene synthase; CYP71AV1: amorphadiene-12-hydroxylase; DBR2: artemisinic aldehyde Δ11(13) reductase; DHAA: dihydroartemisinic aldehyde; DXR: 1-deoxy-D-xylulose 5-phosphate reductase; DXS: 1-deoxy-D-xylulose 5-phosphate synthase; ECS: *epi*-cedrol synthase; FDP: farnesyldiphosphate; FDS: farnesyl diphosphate synthase; GAS: germacrene A synthase; GDP: geranyldiphosphate; HDR: hydroxy-2-methyl-2-(*E*)-butenyl 4-diphosphate reductase; HMGR: 3-hydroxy-3-methyl-glutaryl-CoA reductase; IDP: isopentenyldiphosphate; MSMO: Murashige and Skoog medium with minimal organics; OPR3: 12-oxophytodienoate reductase; PAL: phenylalanine ammonia lyase; RED1: dihydroartemisinic aldehyde reductase; SQS: squalene synthase.

## Authors' contributions

LO planned the experimental setup and PB was involved in the study design. AL prepared the plants. LO and AE carried out the RNA extraction, cDNA synthesis and qPCR analysis and analyzed the data. LO and AL performed the fluorescence microscopy. PB, LO, AL drafted and wrote the manuscript. All authors have read and approved the final manuscript.

## References

[B1] Bosman A Mendis KN A major transition in malaria treatment: The adoption and deployment of artemisinin-based combination therapies Am J Trop Med Hyg 2007 77 193 197 18165492

[B2] Covello PS Making artemisinin Phytochemistry 2008 69 2881 2885 10.1016/j.phytochem.2008.10.001 18977499

[B3] Duke SO Paul RN Development and fine structure of the glandular trichomes of *Artemisia annua *L Int J Plant Sci 1993 154 107 118 10.1086/297096

[B4] Simon JE Charles D Cebert E Grant L Janick J Whipkey A Janick J and Simon JE *Artemisia annua *L.: a promising aromatic and medicinal Advances in new crops 1990 Portland: Timber Press 522 526

[B5] Mercke P Crock J Croteau R Brodelius PE Cloning, expression and characterization of epi-cedrol synthase, a sesquiterpene cyclase from *Artemisia annua *L Arch Biochem Biophys 1999 369 213 222 10.1006/abbi.1999.1358 10486140

[B6] Mercke P Bengtsson M Bouwmeester HJ Posthumus MA Brodelius PE Molecular cloning, expression, and characterization of amorpha-4,11-diene synthase, a key enzyme of artemisinin biosynthesis *in Artemisia annua *L Arch Biochem Biophys 2000 381 173 180 10.1006/abbi.2000.1962 11032404

[B7] Cai Y Jia J-W Crock J Lin Z-X Chen X-Y Croteau R A cDNA clone for β-caryophyllene synthase from *Artemisia annua* Phytochemistry 2002 61 523 529 10.1016/S0031-9422(02)00265-0 12409018

[B8] Bertea CM Voster A Verstappen FW Maffei M Beekwilder J Bouwmeester HJ Isoprenoid biosynthesis in *Artemisia annua*: cloning and heterologous expression of a germacrene A synthase from a glandular trichome cDNA library Arch Biochem Biophys 2006 448 3 12 10.1016/j.abb.2006.02.026 16579958

[B9] Picaud S Brodelius M Brodelius PE Expression, purification and characterization of β-farnesene synthase from *Artemisia annua *L Phytochemistry 2005 66 961 967 10.1016/j.phytochem.2005.03.027 15896363

[B10] Brown GD The biosynthesis of artemisinin and the phytochemistry of *Artemisia annua *L Molecules 2010 15 7603 7698 10.3390/molecules15117603 21030913 PMC6259225

[B11] Bouwmeester HJ Wallaart TE Janssen MH van Loo B Jansen BJ Posthumus MA Schmidt CO de Kraker JW Konig WA Franssen MC Amorpha-4, 11-diene synthase catalyses the first probable step in artemisinin biosynthesis Phytochemistry 1999 52 843 854 10.1016/S0031-9422(99)00206-X 10626375

[B12] Teoh KH Polichuk DR Reed DW Nowak G Covello PS *Artemisia annua *L. (Asteraceae) trichome-specific cDNAs reveal CYP71AV1, a cytochrome P450 with a key role in the biosynthesis of the antimalarial sesquiterpene lactone artemisinin FEBS Lett 2006 580 1411 1416 10.1016/j.febslet.2006.01.065 16458889

[B13] Brown GD Sy LK *In vivo *transformations of artemisinic acid in *Artemisia annua *plants Tetrahedron 2006 63 9548 9566 10.1016/j.tet.2007.06.062

[B14] Brown GD Sy LK *In vivo *transformations of dihydroartemisinic acid in *Artemisia annua *plants Tetrahedron 2004 60 1139 1159 10.1016/j.tet.2003.11.070

[B15] Zhang Y Teoh KH Reed DW Maes L Goossens A Olson DJ Ross AR Covello PS The molecular cloning of artemisinic aldehyde Δ11(13) reductase and its role in glandular trichome-dependent biosynthesis of artemisinin in *Artemisia annua* J Biol Chem 2008 283 21501 21508 10.1074/jbc.M803090200 18495659

[B16] Teoh KH Polichuk DR Reed DW Covello PS Molecular cloning of an aldehyde dehydrogenase implicated in artemisinin biosynthesis in *Artemsia annua* Botany 2009 87 635 642 10.1139/B09-032

[B17] Rydén A-M Identification, characterization and expression of early biosynthetic genes from *Artemisia annua* PhD Thesis 2010 University of Groningen, Department of Pharmaceutical Biology

[B18] Rydén A-M Ruyter-Spira C Quax WJ Osada H Muranaka T Kayser O Bouwmeester H The Molecular cloning of dihydroartemisinic aldehyde reductase and its implication in artemisinin biosynthesis in *Artemisia annua* Planta Med 2010 76 15 1778 1783 20486073 10.1055/s-0030-1249930

[B19] Wallaart TE Pras N Beekman AC Quax WJ Seasonal variation of artemisinin and its biosynthetic precursors in plants of *Artemisia annua *of different geographical origin; proof for the existence of chemotypes Planta Med 2000 66 57 62 10.1055/s-2000-11115 10705736

[B20] Dhingra V Narasu ML Purification and characterization of an enzyme involved in biochemical transformation of arteannuin B to artemisinin from *Artemisia annua* Biochem Biophys Res Commun 2001 281 558 561 10.1006/bbrc.2000.4197 11181083

[B21] Ferreira JFS Janick J Janick J Distribution of artemisinin in *Artemisia annua* Progress in new crops 1996 Arlington: ASHS Press 579 584

[B22] Tellez MR Canel C Rimando AM Duke SO Differential accumulation of isoprenoids in glanded and glandless *Artemisia annua *L Phytochemistry 1999 52 1035 1040 10.1016/S0031-9422(99)00308-8

[B23] Biswas KK Foster AJ Aung T Mahmoud SS Essential oil production: relationship with abundance of glandular trichomes in aerial surface of plants Acta Physiol Plant 2009 31 13 19 10.1007/s11738-008-0214-y

[B24] Chappell J Wolf F Proulx J Cuellar R Saunders C Is the reaction catalyzed by 3-hydroxy-3-methylglutaryl coenzyme A reductase a rate-limiting step for isoprenoid biosynthesis in plants? Plant Physiol 1995 109 1337 1343 12228673 10.1104/pp.109.4.1337 PMC157667

[B25] Schaller H Grausem B Benveniste P Chye M-L Tan CT Song Y-H Chua N-H Expression of the *Hevea brasiliensis *(H.B.K.) müll. Arg. 3-hydroxy-3-methylglutaryl coenzyme A reductase 1 in tobacco results in sterol overproduction Plant Physiol 1995 109 761 770 12228630 10.1104/pp.109.3.761 PMC161375

[B26] Ram M Khan MA Jha P Khan S Kiran U Ahmad MM Javed S Abdin MZ HMG-CoA reductase limits artemisinin biosynthesis and accumulation in *Artemisia annua *L. plants Acta Physiol Plant 2010 32 859 866 10.1007/s11738-010-0470-5

[B27] Stermer BA Bianchini GM Korth KL Regulation of HMG-CoA reductase activity in plants J Lipid Res 1994 35 1133 1140 7964176

[B28] Cunillera N Boronat A Ferrer A Spatial and temporal patterns of GUS expression directed by 5' regions of the *Arabidopsis thaliana *farnesyl diphosphate synthase genes FPS1 and FPS2 Plant Mol Biol 2000 44 747 758 10.1023/A:1026588708849 11202437

[B29] Han JL Liu BY Ye HC Wang H Li ZQ Li GF Effects of overexpression of the endogenouse farnesyl diphosphate synthase on the artemisinin content in *Artemisia annua *L J Integr Plant Biol 2006 48 482 487 10.1111/j.1744-7909.2006.00208.x

[B30] Banyai W Kirdmanee C Mii M Supaibulwatana K Overexpression of farnesyl pyrophosphate synthase (FPS) gene affected artemisinin content and growth of *Artemisia annua *L Plant Cell Tiss Organ Cult in press

[B31] Matsushita Y Kang WK Charlwood BV Cloning and analysis of a cDNA encoding farnesyl diphosphate synthase from *Artemisia annua* Gene 1996 172 207 209 10.1016/0378-1119(96)00054-6 8682304

[B32] Zhao Y Ye H Li G Chen D Liu Y Cloning and enzymology analysis of farnesyl pyrophosphate synthase gene from a superior strain of *Artemisia annua *L Chi Sci Bull 2003 48 63 67

[B33] Cordoba E Salmi M León P Unravelling the regulatory mechanisms that modulate the MEP pathway in higher plants J Exp Bot 2009 60 2933 2943 10.1093/jxb/erp190 19584121

[B34] Schramek N Wang H Römisch-Margl W Keil B Radykewicz T Winzenhörlein B Beerhues L Bacher A Rohdich F Gershenzon J Liu B Eisenreich W Artemisinin biosynthesis in growing plants of *Artemisia annua*. A ^13^CO_2 _study Phytochemistry 2010 71 179 187 10.1016/j.phytochem.2009.10.015 19932496

[B35] Chang Y-J Song S-H Park S-H Kim S-U Amorpha-4,11-diene synthase of *Artemisia annua: *cDNA isolation and bacterial expression of a terpene synthase involved in artemisinin biosynthesis Arch Biochem Biophys 2000 383 178 184 10.1006/abbi.2000.2061 11185551

[B36] Wallaart TE Bouwmeester HJ Hille J Poppinga L Maijers NC Amorpha-4,11-diene synthase: cloning and functional expression of a key enzyme in the biosynthetic pathway of the novel antimalarial drug artemisinin Planta 2001 212 460 465 10.1007/s004250000428 11289612

[B37] Li ZQ Liu Y Liu BY Wang H Ye HC Li GF Cloning, *E. coli *expression and molecular analysis of amorpha-4,11-diene synthase from a high-yield strain of *Artemisia annua *L J Integ Plant Biol 2006 48 1486 1492 10.1111/j.1744-7909.2006.00381.x

[B38] Ro DK Paradise EM Ouellet M Fisher KJ Newman KL Ndungu JM Ho KA Eachus RA Ham TS Kirby J Chang MC Withers ST Shiba Y Sarpong R Keasling JD Production of the antimalarial drug precursor artemisinic acid in engineered yeast Nature 2006 440 940 943 10.1038/nature04640 16612385

[B39] Olsson ME Olofsson LM Lindahl A-L Lundgren A Brodelius M Brodelius PE Localization of enzymes of artemisinin biosynthesis to the apical cells of glandular secretory trichomes of Artemisia annua L Phytochemistry 2009 68 1864 1871 10.1016/j.phytochem.2009.07.009 19664791

[B40] Schaller F Biesgen C Müssig C Altmann T Weiler EW 12-Oxophytodienoate reductase 3 (OPR3) is the isoenzyme involved in jasmonate biosynthesis Planta 2000 210 979 984 10.1007/s004250050706 10872231

[B41] Maes L Van Nieuwerburgh FCW Zhang Y Reed DW Pollier J Vande Casteele SRF Inzé D Covello PS Deforce DLD LD Goossens A Dissection of the phytohormonal regulation of trichome formation and biosynthesis of the antimalarial compound artemisinin in *Artemisia annua *plants New Phytologist 2011 189 176 189 10.1111/j.1469-8137.2010.03466.x 20874804

[B42] Livak KJ Schmittgen TD Analysis of relative gene expression data using real- time quantitative PCR and the 2^-ΔΔ*C*T ^method Methods 2001 25 402 408 10.1006/meth.2001.1262 11846609

[B43] Liu CZ Wang YC Ouyang F Ye HC Li GF Production of artemisinin by hairy root cultures of *Artemisia annua *L Biotechnol Lett 1997 19 927 929 10.1023/A:1018362309677

[B44] Weathers PJ Bunk G McCoy MC The effect of phytohormones on growth and artemisinin production in *Artemisia annua *hairy root In Vitro Cell Dev Biol - Plant 2005 41 47 53 10.1079/IVP2004604

[B45] Banerjee S Zehra M Gupta MM Kumar S *Agrobacterium rhizogenes*-mediated transformation of *Artemisia annua*: production of transgenic plants Planta Med 1997 63 467 469 10.1055/s-2006-957737 17252369

[B46] Ma C Wang H Lu X Wang H Xu G Liu B Terpenoid metabolic profiling analysis of transgenic *Artemisia annua *L. by comprehensive two-dimensional gas chromatography time-of-flight mass spectrometry Metabolomics 2009 5 497 506 10.1007/s11306-009-0170-6

[B47] Zhang D Jennings SM Robinson GW Poulter CD Yeast squalene synthase: Expression, purification and characterization of soluble recombinant enzyme Arch Biochem Biophys 1993 304 133 143 10.1006/abbi.1993.1331 8323279

[B48] Liu CZ Ye HC Wang H Li GF Molecular cloning, *Escherichia coli *expression and genomic organization of squalene synthase gene from *Artemisia annua* Acta Bot Sin 2003 45 608 613

[B49] Zhang L Jing F Li F Li M Wang Y Wang G Sun X Tang K Development of transgenic *Artemisia annua *(Chinese wormwood) plants with an enhanced content of artemisinin, an effective anti-malarial drug, by hairpin-RNA-mediated gene silencing Biotechnol Appl Biochem 2009 52 199 207 10.1042/BA20080068 18564056

[B50] Ferreira JFS Gonzalez JM Analysis of underivatized artemisinin and related sesquiterpene lactones by high-performance liquid chromatography with ultraviolet detection Phytochem Anal 2009 20 91 97 10.1002/pca.1101 18980258

[B51] Ruijter JM Ramakers C Hoogaars WMH Karlen Y Bakker O van den Hoff MJB Moorman AFM Amplification efficiancy, linking baseline and bias in the analysis of quantitative PCR data Nucl Acids Res 2009 37 e45 10.1093/nar/gkp045 19237396 PMC2665230

[B52] Pfaffl MW Tichopád A Prgomet C Neuvians TP Determination of stable housekeeping genes, differentially regulated target genes and sample integrity: *BestKeeper *- Excel-based tool using pair-wise correlations Biotechnol Lett 2004 26 509 515 10.1023/B:BILE.0000019559.84305.47 15127793

[B53] Pfaffl MW Horgan GW Dempfle L Relative expression software tool (REST) for group-wise comparison and statistical analysis of relative expression results in real-time PCR Nucl Acids Res 2002 30 e36 10.1093/nar/30.9.e36 11972351 PMC113859

[B54] Picaud S Olofsson L Brodelius M Brodelius PE Expression, purification and characterization of amorpha-4,11-diene synthase from *Artemisia annua *L Arch Biochem Biophys 2005 436 2 215 226 10.1016/j.abb.2005.02.012 15797234

